# Academy of Stomatology

**Published:** 1903-06

**Authors:** 

**Affiliations:** Academy of Stomatology


### Reports of Society Meetings.

### ACADEMY OF STOMATOLOGY.

A REGULAR meeting of the Academy of Stomatology was held at its rooms, 1731 Chestnut Street, Philadelphia, on Tuesday evening, December 23, 1902, the President, Dr. R. H. D. Swing, in the chair.

The following papers were read and discussed: "Some Considerations pertaining to Dental Diagnosis," by Dr. John C. Curry, of the Academy; "Baked Porcelain Restorations of Broken Bridge Facings," by Dr. Joseph E. Duffield, of the Academy.

(For Dr. Curry's paper, see page 349. For Dr. Duffield's paper, see page 409.)

### DISCUSSION ON DR. J. C. CURRY'S PAPER.

*Dr. Otto E. Inglis.*—Conan Doyle put words into the mouth of Sherlock Holmes to the effect that to be a detective one must have imagination. I feel that this is true of diagnosis. One must see with his physical eye what can be seen, and with his mind's eye what cannot be seen but may be possible. To jump to the complex or uncertain, overlooking the simplicities, is to make a grave mistake when a dental diagnosis is to be made. At the same time, not to possess a fairly complete knowledge of possibilities in and about the parts, or of means or appliances to test these possibilities, when a diagnosis is not easily made, is to fail to distinguish the disease present. Dr. Garretson was wont to say that conditions commonly tell all about themselves, and that all that is necessary is to recognize the signs by which they express themselves. During the animated discussion of the uric acid diathesis by the profession a few years ago a student consulted a dentist in relation to his left upper central incisor, which was elongated and slightly sore. Diathesis was descanted upon and antigout remedies suggested. As a matter of fact, apical abscess due to a septic pulp existed, and was later diagnosed at a distance of six feet, simply by the color of the tooth and the associated symptoms. This illustrates the overlooking of the simplicities.

The greatest difficulty in the way of diagnosis of diseases of dental origin is the reflex character of the pains and the presence of a number of possible causes of the pain. Isolation and testing by appropriate tests is the rational method of procedure.

As the essayist has said, a few well-directed questions will often elicit valuable information; as, for example, Where is the pain located? Do you have it during mastication? Does any tooth feel longer than the rest or painful when touched? Do salt, sweet, or acid substances produce a steady pain? Does the contact of hot or cold liquids produce pain; if so, how long does it last and is it a steady or a throbbing pain, and is the pain greatest when heat is applied, or *vice versa?*

I listen to the patient's description of sensations only for the purpose of obtaining a clue, while I follow up with some such question. If, for example, the patient states that pain is elicited upon touching the cervix of a tooth with the finger-nail, I think of exposed and hypersensitive dentine and investigate accordingly, touching the part and endeavoring to repeat the experience of the patient.

If the patient states that pain is felt at such a cervix when a breath of air is inhaled, and I find a deep and sensitive cavity near by, I ignore the patient's explanation and investigate by isolation test, etc.

In short, the simple and rational causes are first looked for, and if not found I begin to look beneath the surface with my mind's eye, and to think of crevicing leakage, septic dentine beneath fillings, loose fillings masticated upon, thermal shocks transmitted by fillings, hyperæmias, inflammation or abscess of the pulp, pulp degeneration or its constructive activity and also of perfect or imperfect canal fillings, dead pulps, septic or aseptic irritation of apical tissues, roots broken off and left in the process, marginal or destructive pericementitis, and so on. Each is excluded in order of probability. Where no cause is discoverable in the teeth present which may produce the symptoms, reference is made to teeth in process of eruption or which are impacted in the jaw. Next, the parts associated with the teeth are looked into. Each pathological condition has its usual train of characteristic symptoms, as well as some that are common to many dental diseases.

It is not to be supposed that one may not err in the exact diagnosis at first. A patient may have tenderness to tapping, a sensation of fulness and throbbing at the apical space, and an absence of dentinal sensitivity either to cutting or thermal changes, and even some opacity of the tooth-crown, all of which are associated with an incipient apical abscess, and yet upon opening the tooth an abscess of the pulp may be found. Usually in such a case there is a greater reaction to heat than to cold, while sudden onsets and remissions or intermissions of the lancinating pain are not uncommon.

The obscure pathologies, such as hypercementosis, absorption of roots, and pulp nodules may at times be inferred from symptoms, but their diagnosis without the X-ray is often only to be inferrred by exclusion of other more common causes.

Failing a dental explanation of oral diseases or natural explanation of dental disease, local oral condition and then systemic disturbances are to have consideration in the light of professional or personal experience with such possibilities, and, failing these, the new experience should be recorded for the benefit of our brethren.

Search for primary causes is often attended with difficulty, either because of the fact that the patient is ignorant of the history of the case or mendacious. I have in mind a case of longitudinal fracture of a sound molar, in regard to which I questioned a lady. She denied biting upon hard substances, but her daughter the next day laughed and said, "Why mother is continually biting nuts and other hard substances."

In cases of erosion a gouty history may be denied by the patient. The essayist has brought out some interesting points to be considered in making up a diagnosis, which, however, seems not to require any comment.

*Dr. James Truman.*—The subject has been so thoroughly treated by the essayist that there is very little to say. I am very glad, however, that the paper has come before the society. The author passes over rapidly the subject of the first dentition, and with equal rapidity considers the permanent dentition. Now, I take it that comparatively few have observed the diagnostic importance of conditions that occur in the development or eruption of the permanent teeth. Take, for instance, the second molar. Except in a very recent publication, no one has alluded to the second molar as an important factor in diagnosis. Several years ago I called the attention of the profession to the cerebral disturbance that may occur during the eruption of this tooth. At the ninth year it assumes the vertical position and may impinge upon the inferior dental nerve, and the reaction upon the pulp produces many nervous disturbances. I have also frequently found reflex disturbances manifested in the ear and other parts of the body arising from this tooth. The remedy is very simple, and consists in relieving the pressure upon the pulp by lancing deeply through the gum. Then again, in speaking of the eruption of the third molar the author does not describe the diagnostic signs. When it is lying horizontally in the jaw, how is the dentist to know it is there? He cannot see it. It is not visible. There are certain violent reflex pains which appear, and the dentist will have to judge of their cause by the number of teeth in the mouth. If there be but one molar, and there be a large space left by the extraction of the first molar, it is possible that the third may not be in a horizontal position. If both the first and second molars be in place, the dentist can diagnose almost to a certainty that there is a tooth pressing against the distal surface of the roots of the second molar tooth and causing reflex disturbance.

The author alludes to arsenical necrosis as if it were a very easy thing to diagnose. I do not quite so regard it. In the teaching of the undergraduates I have not found this a simple matter to deal with. Arsenical necrosis is practically the same as that produced by any other inflammation, with the added toxic effect of the agent.

On the whole, I like the paper, but I wish the author had gone a little farther into many matters that might be of advantage to those who need just that kind of instruction.

*Dr. J. A. Woodward.*—I think it is a safe rule, that when making a diagnosis one cannot clearly see his way, not to do anything, but simply to wait until the developments make the cause plain. I remember a case, reported by some one in the West, of a man who apparently had an irritated pulp in one of the upper bicuspid teeth. He tried in every way to save that pulp, but could not relieve his patient. He destroyed the pulp, but without relief. He was considering the advisability of destroying the pulp of the adjoining tooth, when the man was attacked by a very severe irritation of the skin of his whole head and face. Then the irritation of the tooth subsided. This was simply a skin irritation first felt in the tooth. So it is always better, in obscure cases, to wait until one knows clearly what to do before acting.

*Dr. S. H. Guilford.*—Professor Garretson used to say that in diagnosing a case he did it by exclusion. That is to say, he would conclude that in a part there might be a certain number of different pathological conditions. He would go over the case carefully, excluding in turn each condition as not indicated by the symptoms, until he came to the last, which he claimed must be present and must fit the symptoms. In a general way, that is good reasoning in diagnosis, yet I do not suppose it would always be efficient. There is an old adage which says that when you don't know what to do, do nothing. Still, sometimes I think the dentists have, like the physicians, to do something to satisfy the patient when they do not know exactly what to do.

Speaking of hypercementosis, I recall a case of a lady whose teeth were in good order, but she came to me complaining of neuralgic pain, and in the description of the case stated that whenever she was exposed to the night air she was apt to have the symptoms come on. I could find no fault with the teeth, and concluded that there must be some hidden irritation. I partially located it in the lower third molars, and persuaded her to have one of them removed. This gave her partial relief and the extraction of the other was followed by entire relief. Both were greatly enlarged at the roots. Cases of that kind we all see frequently, and they are the most difficult to diagnose. Sometimes the pain will localize in a certain tooth, but sometimes it does not, and then we are at a loss to know what to do. It may not be a serious thing to drill into a tooth and devitalize it, but it always seems to me a serious thing to do, and for that reason I hesitate in all such cases.

*Dr. Curry* (closing).—In answer to Dr. Truman, I would say that when asked to read a paper I was given to understand that it should be of a certain length and occupy a certain time, and I spoke as fully upon the matter of dentition as I could in the time allowed.

In regard to Dr. Woodward's remarks, I am a stickler for conservatism. I do not often act in a case until I am reasonably sure of the diagnosis. The title of the paper is suggestive, and the things spoken of are some considerations pertaining to diagnosis, not all the considerations pertaining to all the points of diagnosis. Dr. Guilford calls attention to pain usually described as "neuralgic," by which we understand the pain described by the patient as of that vague, ill-defined character, and which shoots here and there, varying in intensity according to the cause, but which we all recognize as being a different kind of pain from the nerve-ache, or soreness or general tenderness.

I thank the society for the discussion and for the very liberal treatment accorded me.

DISCUSSION ON DR. DUFFIELD'S PAPER.

*Dr. S. H. Guilford.*—I am glad to have seen this work of Dr. Duffield's, which strikes me as something new and of decided value. We have had a great many methods presented to us of repairing bridges and of putting in new porcelain facings. For my own part, at the next opportunity I shall give this method a trial. Some one has said there is nothing so weak as a tooth with two pins in it, because each pin is an element of weakness, and with this little box the tooth is stronger than with two pins. More than that, the box requires no special tooth. It may be made concave to fit over the pins, and that is all we require. It is a very excellent method so far as I can judge.

*Dr. Duffield.*—I have probably twelve or fifteen repairs of this character, which, as I said in my paper, have been in use nearly three years. Out of that number, I think I have had one failure, in which case the bite was close. I think had the tooth been soldered it would have broken off just the same. I think that you will find not only that this will save a great deal of labor, but that you will be able to make a repair which in adaptation and in general effect is far superior to the old method.

Adjourned.

OTTO E. INGLIS,
*Editor Academy of Stomatology.*

